# Supplementary material for: Learning curve and surgical outcome of robotic assisted colorectal surgery with ERAS program
Source: Sci Rep. 2022 Nov 29;12:20566. doi: 10.1038/s41598-022-24665-w (PMC9709162; doi:10.1038/s41598-022-24665-w)
Supplement: Supplementary file 2 — Supplementary Information 2. [file 41598_2022_24665_MOESM2_ESM.zip › RAL Raw data-Ñ[▒K/094/HS_ñw╝╨░O▒Kñσ.pdf]

正式摘要(電子簽章：000000220194667)<br>W52 / W52 ☐英文標題(注意:無英文姓名-請補病人基本檔!)	☐【讀取】憑證	

臺中榮民總醫院
出院病歷摘要

轉入號： 轉出號：

[redacted]

(8)流水編號	
(9)入院日期	110/06/30 (2021) 直腸外科 W52 - 072
(10)轉科日期	
(11)出院日期	110/07/05 (2021) 住院天數計 5 日

診斷

- 入院:1. Rectal adenocarcinoma, 15cm FAV
  2. FL, stage 4, w/ BM involvement, s/p C/T w/ R-CHOP, C8, w/ rituximab maintenance therapy (1080220), w/ relapse (POD24) (by PET on 1080410)
  3. HTN under medication
  4. DM, Type 2 under medication
  5. Rt kidney atrophy
  6. Arrhythmia with frequent PVCs with bigeminal and trigeminal episodes w/o VT, intermediate frequency of PACs, under Bokey, Bisoprolol, Hydralazine
- 出院:1. Rectal adenocarcinoma, 15cm FAV, s/p Robotic LAR on 7/01
  2. FL, stage 4, w/ BM involvement, s/p C/T w/ R-CHOP, C8, w/ rituximab maintenance therapy (1080220), w/ relapse (POD24) (by PET on 1080410)
  3. HTN under medication
  4. DM, Type 2 under medication
  5. Rt kidney atrophy
  6. Arrhythmia with frequent PVCs with bigeminal and trigeminal episodes w/o VT, intermediate frequency of PACs, under Bokey, Bisoprolol, Hydralazine

主訴

- Rectal Ca, adenocarcinoma, 15cm FAV

病史

- This 55y/o male has hx of
  1. FL, stage 4, w/ BM involvement, s/p C/T w/ R-CHOP, C8, w/ rituximab maintenance therapy (1080220), w/ relapse (POD24) (by PET on 1080410)
  2. HTN under medication
  3. DM2 under medication
  4. Rt kidney atrophy
  5. r/o idiopathic VPC, under medications

  According to him, he received healthy checkup and positive stool occult. Then, he was referred to CRS OPD. Colon scopy disclosed 3 polyps at 13, 15, 35cm FAV and pathological report revealed polyp, 15cm FAV, adenocarcinoma, moderately differentiated, arising in tubulovillous adenoma. After discussion, surgical intervention was suggested and he accepted it.
  This time, he was admitted for scheduled robotic LAR.

體檢發現

-
  - General appearance :
    - easy looking , conscious clear , without cardiopulmonary distress
    - Vital signs自動帶入date 體溫(BT) 脈搏(P) 呼吸(R) 血壓(BP) 身高(BH) 體重(BW) BMI
      20210630 36.3℃ 75次/分 19次/分 148/89mmHg 167.1cm 102.9kg 36.9
    - Vital signs: B.P. 146/76 mmHg , P.R. 70 /min , R.R. 18 /min , B.T. 36 ℃
  - HEENT :
    - Eyes: no anemic conjunctiva, no icteric sclera, normal EOM
    - ENT: no otorrhea, no rhinorrhea, no erythematous mucosa, no injected tonsil, no deformity
  - Neck: supple, freely movable, trachea midline,no deformity, no engorged jugular vein , no palpable thyroid or lymph node enlargement
  - Chest:
    - Inspection: Grossly normal, symmetric and fully expansion, no accessory muscle used, no operation scar, no spider angiomata, no gynecomastia
    - Palpation: no palpable mass or nodule, no subcutaneous emphysema, no tenderness
    - Ausculation: clear breathing sound
  - Heart:
    - Palpation: no thrill, no heave
    - Percussion: normal heart size
    - Ausculation: regular heart beat, normal S1, S2, no gallop, no murmur
  - Extremities: No deformity, full ranged of motion without limitation, no clubbing finger or nail cyanosis, no pitting edema, no ulcer or pigmentation, no obvious skin lesion, no varicose vein, no bone, joint or muscle

- tenderness

CRS CONDITION

- Karnofsky performance status: (100) Normal, no complaints, no evidence of disease
- PE of Abdomen:
- CEA: .33 ng/ml
- Imaging Studies:
- Colonoscope report: The sedative colonscopy was done at 3F OP room. The scope was done up to cecum, 90 cm FAV smoothly. Few watery stool retension. No obvious diverticulum. A polyp s/p hot snare polypectomy at 35cm FAV. Another polyp s/p hot snare polypectomy with 3 hemo-clip applied at 15cm FAV. The other polyp s/p hot snare polypectomy with 1 hemo-clip applied at 13cm FAV. Hemorrhoid.
- Figures:
  1. colon & rectum

手術日期及方法

- 手術日期及方法20210701 Robot Assisted Low Anterior Resection

住院治療經過

- After admission, the patient received Robotic LAR on 2021/07/01. The patient was under post-op wound care and pain control. Transamin and Vitamin K were given due to easily oozing intra-operatively and coagulopathy, suspected anticoagulant related. On ERAS protocol, Foley's catheter was removed on 7/02. Penrose drain was removed on 7/04. Under relative stable condition, the patient was discharged with OPD follow up.

合併症

一般檢查

- 累積報告 :

DATE	TIME	ALB	TP	BIL.T	BIL.D	ALKP	AST	ALT	LDH	AMY	LIPASE	NH3	Ferritin	UA
1100630	1641	4.1		0.7	0.2		41	22						
1100705	0845	3.8		1.17			35	16						

DATE	TIME	WBC	RBC	HGB	HCT	MCV	PLT	BLST	NEUT	BAND	SEG	LYM	MON	EOS	BAS
1100630	1640	3770	4.10	12.1	36.5	89.0	53		70.6			15.6	9.3	3.2	1.3
1100701	0547						63								
1100701	1759	3840		11.2	33.0		60		84.1			10.2	4.7	0.5	0.5
1100702	1054	3930	3.73	11.3	32.7	87.7	72		74.8			14.2	8.9	1.3	0.8
1100705	0859	3550		12.4	36.7	89.7	69		67.3			17.5	9.9	4.2	1.1

DATE	TIME	NA	K	CL	BUN	CREAT	eGFR	CA	Mg	P	CRP	ESR	PCT	D-Dimer	LACTATE
1100630	1641	139	4.3		17	1.16		8.7							
1100701	1759	138	4.7			1.24	64.09	8.0							
1100705	0845	139	4.2		15	1.21	65.93	8.3							

DATE	TIME	GLUCOSE	HbA1C	CHOL	TG	HDL	LDL	KETONE	CK	CKMB	TRO-T	PT-P	APTT-P	NT-pro BNP
1100701	1800											11.6		
1100705	0857											10.6		

DATE	TIME	PH	BEB	SCVO2	PO2	HCO3	HGB	PCO2	TCO2
1100705	0806	7.358	0.8	38.6	23.8	27.1		49.2	28.6

特殊檢查

病理報告

- Pending

其它

出院時情況

- 3.改門診治療

出院指示

藥名	劑量	劑量單位	用法	頻次	數量	數量單位	現況	天數	單位
Ulstop tab 20mg	1	TAB	PO	BID	6	TAB	TH	3	天
Mag. Oxide tab	2	TAB	PO	TID	48	TAB	TH	8	天
Ultracet tab	1	TAB	PO	Q6H	32	TAB	TH	8	天
Spironolactone-25	1	TAB	PO	BID	16	TAB	TH	8	天

出院計劃

- 門診日期
  - date on 2021/07/13
- Pending pathological report. Treatment plan to be discuss.

主治醫師 2062F 蔣鋒帆 / 住院醫師 2345B 陳映晴

正式摘要(電子簽章：000000220194667)

W52 / W52

doctorRank:B
